# Effective Tumor Targeting by EphA2-Agonist-Biotin-Streptavidin Conjugates

**DOI:** 10.3390/molecules26123687

**Published:** 2021-06-17

**Authors:** Parima Udompholkul, Carlo Baggio, Luca Gambini, Yu Sun, Ming Zhao, Robert M. Hoffman, Maurizio Pellecchia

**Affiliations:** 1 Division of Biomedical Sciences, School of Medicine, University of California Riverside, 900 University Avenue, Riverside, CA 92521, USA; pudom001@ucr.edu (P.U.); carlo.baggio@medsch.ucr.edu (C.B.); lucaga@ucr.edu (L.G.); 2 AntiCancer Inc., 7917 Ostrow St., San Diego, CA 92111, USA; sy831225@gmail.com (Y.S.); mzhao@anticancer.com (M.Z.); all@anticancer.com (R.M.H.); 3 Department of Surgery, University of California, 9300 Campus Point Dr #7220, La Jolla, San Diego, CA 92037, USA

**Keywords:** agonistic EphA2 peptides, streptavidin, pancreatic cancer, breast cancer, cancer imaging, orthotopic cancer models

## Abstract

We recently reported on a potent synthetic agent, 135H11, that selectively targets the receptor tyrosine kinase, EphA2. While 135H11 possesses a relatively high binding affinity for the ligand-binding domain of EphA2 (Kd~130 nM), receptor activation in the cell required the synthesis of dimeric versions of such agent (namely 135H12). This was expected given that the natural ephrin ligands also need to be dimerized or clustered to elicit agonistic activity in cell. In the present report we investigated whether the agonistic activity of 135H11 could be enhanced by biotin conjugation followed by complex formation with streptavidin. Therefore, we measured the agonistic EphA2 activity of 135H11-biotin (147B5) at various agent/streptavidin ratios, side by side with 135H12, and a scrambled version of 147B5 in pancreatic- and breast-cancer cell lines. The (147B5)_n_-streptavidin complexes (when *n* = 2, 3, 4, but not when *n* = 1) induced a strong receptor degradation effect in both cell lines compared to 135H12 or the (scrambled-147B5)_4_-streptavidin complex as a control, indicating that multimerization of the targeting agent resulted in an increased ability to cause receptor clustering and internalization. Subsequently, we prepared an Alexa-Fluor-streptavidin conjugate to demonstrate that (147B5)_4_-AF-streptavidin, but not the scrambled equivalent complex, concentrates in pancreatic and breast cancers in orthotopic nude-mouse models. Hence, we conclude that these novel targeting agents, with proper derivatization with imaging reagents or chemotherapy, can be used as diagnostics, and/or to deliver chemotherapy selectively to EphA2-expressing tumors.

## 1. Introduction

The receptor tyrosine kinase of the Eph family controls various cellular functions including cell–cell communications that are mediated by their interactions with cell surface ephrin ligands [1,2,3,4,5,6,7,8,9,10,11,12,13,14,15,16]. In cancer cells, the EphA2 receptor subtype is pro-oncogenic when not bound to its ligands, where it promotes angiogenesis, cell migration, and increases metastases. Aberrant EphA2 overexpression causes an excess of unbound receptor and it contributes to the spread of certain solid tumors including prostate cancer [1,17], melanoma [13,18], breast cancer [2,3], brain cancer [10,19], ovarian cancer [20], urinary bladder cancer [11], pancreatic cancer [5,6,21,22], esophageal cancer [23], lung cancer [24], stomach cancer [25], and several types of leukemia [15,16]. In pancreatic cancer, an extremely aggressive tumor which accounts for about 7% of all cancer deaths in the United States, EphA2 overexpression is inversely correlated with patient survival [5,21]. More recent studies underlined the role of EphA2 in driving therapy-resistant pancreatic adenocarcinomas, suggesting that EphA2-targeting agents should be developed and used in combination with current therapeutics [26]. In addition, our recent studies in a variety of pancreatic cancer cell lines, or primary pancreatic cancer tissues, demonstrated elevated EphA2 levels in all studied cases [22].

Similarly, EphA2 is expressed in breast cancer tissue, and its elevated expression correlates with poor patient survival, particularly in triple-negative breast cancer (TNBC). TNBCs are characterized by a lack of hormone receptor (estrogen and progesterone nuclear receptors) expression, as well as a lack of the human epidermal growth factor receptor-2 (HER2), for which therapeutic targeting strategies are available. Currently, TNBC represents one of the most aggressive forms of breast cancer, and disproportionally affects young pre-menopausal women and women of African descent, leaving these patients with limited therapeutic options. Although the EphA2 receptor has been identified as a clinically-relevant biomarker and potential target for TNBC [27], the development of effective diagnostics and therapeutics for these aggressive forms of cancers is currently hampered by the lack of viable pharmacological tools. 

Because ligand-bound EphA2 turns the oncogenic receptor into a tumor suppressor, the design of ephrin mimetics, hence synthetic agonistic EphA2-targeting agents, represents a potentially therapeutically-viable strategy. Recently we reported on synthetic agonistic peptides that by selectively engaging with the EphA2 ligand-binding domain, cause internalization of the receptor, followed by its lysosomal degradation [28]. We recently demonstrated that these agonistic agents strongly reduced BxPc3 pancreatic-cancer cell migration [29], and suppressed tumor metastases in an orthotopic model of EphA2-driven aggressive prostate cancer [30]. Because the agonistic agent causes receptor internalization, we have also demonstrated that when properly conjugated with cytotoxic drugs, EphA2-agonistic agents could serve as peptide–drug conjugates to deliver chemotherapy to EphA2-expressing tumors [22,28,31,32,33,34]. For example, we previously demonstrated that synthetic EphA2-targeting agonistic agents conjugated with gemcitabine or paclitaxel had superior efficacy compared to gemcitabine or paclitaxel alone in mouse models of pancreatic cancer [22] and breast cancer, respectively [31]. 

Using an EphA2 internalization assay in cancer cell lines we recognized that, similar to the ephrin ligands, dimeric versions of these synthetic agents possessed greatly increased cellular efficacy in causing receptor degradation, compared to their monomeric versions. 

Based on the results summarized above, we determined in the present study that multimeric versions of EphA2 agonistic peptides can be obtained by biotin derivatization and conjugation with streptavidin. We report that these streptavidin-based EphA2 targeting agents induce superior receptor degradation in triple-negative MDA-MB-231 breast cancer cells and BxPC3 pancreatic-cancer cells. Moreover, we show that when conjugated with fluorescently-tagged streptavidin, a biotinylated agonist, but not its scrambled control, targets breast and pancreatic cancers in orthotopic mouse models. The present studies will enable future design of EphA2-targeting streptavidin conjugates for diagnostic and therapeutic purposes. 

## 2. Results and Discussion

### 2.1. Design and Characterization of Streptavidin-Based EphA2 Targeting Agents

**Figure 1:**
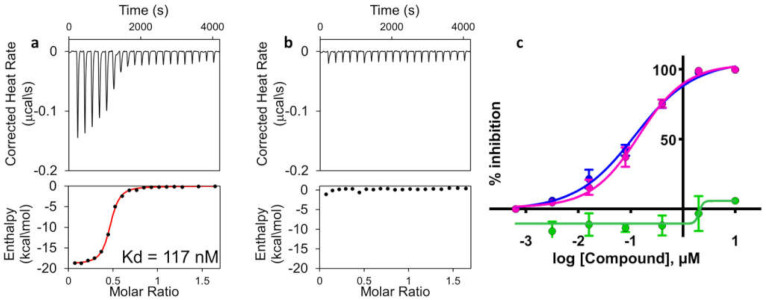
Affinity of EphA2-LBD-targeting agents as detected by ITC (panels A and B) or DELFIA (panel C). (**a**) ITC curve for agent 147B5 for EphA2-LBD (K_d_ = 117 nM). (**b**) ITC curve for the scrambled version of 147B5 against EphA2-LBD, no binding detected. (**c**) DELFIA dose–response curves for agents 135H11 (purple, IC_50_ = 130 nM), 135H12 (blue, IC_50_ = 150 nM), or the scrambled version of 135H11 (green, no inhibition).

We have recently reported on the design of novel EphA2 targeting agonistic ephrin mimetics consisting of 12-mer peptides, enabling the synthesis of agent 135H11 (Table 1) [35]. To characterize the binding properties of the agents, we developed a heterogeneous assay based on the Dissociation-Enhanced Lanthanide Fluorescent Immunoassay (DELFIA) [35] platform, where an earlier-generation biotinylated EphA2-binding peptide is captured on the surface of streptavidin-coated 96-well plates. Subsequently, a 6xHis-EphA2 ligand-binding domain and a fluorescent Europium-conjugated anti-6xHis antibody are added to each well of the 96-well plate, together with a new test agent at various doses. After an incubation time, followed by washing steps, residual fluorescence is measured and correlated with the ability of a given test agent to displace the binding between EphA2-LBD and the reference peptide [35]. Using the DELFIA assay, an IC_50_ value for 135H11 of ~130 nM was determined (Table 1). In the same assay, the scrambled version of 135H11 was inactive (Figure 1). For the current studies with agents that were biotinylated (145B5 and its scrambled version; Table 1), we could not run the DELFIA assay as the agents would be captured by the streptavidin-coated plate, hence we opted to measure direct binding affinities between the agents and EphA2-LBD via isothermal titration calorimetry (ITC). Hence, binding affinities of 147B5 or its scrambled version were obtained by reverse ITC titration with purified EphA2-LBD. In this assay we found a dissociation constant for 147B5 of 117 nM, while no appreciable binding was detected for its scrambled version (Figure 1). 

**Figure 2:**
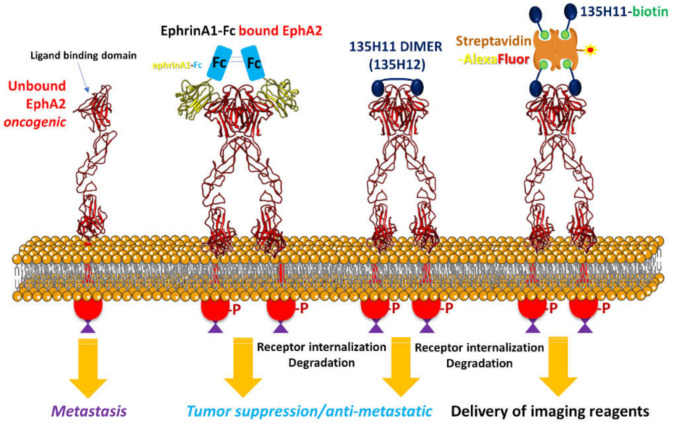
Schematic representation of EphA2 receptor dimerization by agonistic agents. The structures of the EphA4 subtype were used for schematic illustration (PDB IDs 4M4P and 4M4R were used for the apo and ephrinA5 bound, respectively) [38]. Dimeric agents ephrinA1-Fc and 135H12 can efficiently induce dimer formation and receptor activation, internalization, and degradation. The present study evaluates the biotin–streptavidin system (far right) to affect receptor internalization and for its application for imaging and targeted cancer chemotherapy.

In cellular assays, monomeric peptide mimetics, much like the ephrin ligands, displayed limited agonistic activity, measured by observing either receptor phosphorylation or total receptor degradation. Receptor activation with 135H11, or earlier generation agonistic peptides, could be observed only after exposing cells to very high concentrations of ligand (>100 μM) [5,29,31,35,36,37]. This agrees with the observed activity of isolated ephrin ligands, that, even if they have binding affinities in the nanomolar range against EphA2, they are not effective as agonists in cellular assays in monomeric form. In contrast, a dimeric ephrinA1-Fc chimera (Figure 2) is dramatically more effective as an agonist [5] in inducing receptor degradation at nanomolar concentrations [28,29,30,31,34]. Similarly, a previous study, with an earlier and less potent agonistic peptide of sequence SWLAYPGAVSYR, reported that when the agent was dimerized at the C-terminus by an aminoexanoic acid linker, the resulting dimeric peptide was much more potent than its monomer in activating the receptor in the cell [36].

It is likely that the dramatically-increased activity of the dimers in cells is due to their ability to catalyze receptor dimerization and subsequent clustering, triggering internalization and degradation via the lysosome [31,35]. Based on these observations, we recently reported on the synthesis and cellular evaluation of dimeric versions of 135H11, leading to agent 135H12 (Table 1). Of note is that the agents are not cytotoxic for cancer cell lines even when tested at 100 μM, but by causing EphA2 degradation, they limit cell-migratory properties and inhibit metastases in vivo [29,30,31,35].

In the present report we explored the opportunity to obtain multifunctional EphA2-targeting multimers using the biotin–streptavidin system (Figure 2). Hence, we synthesized a biotinylated 135H11 and its scrambled version as a control (Table 1), following the general solid-phase synthetic protocols we have recently reported [35].

**Figure 3:**
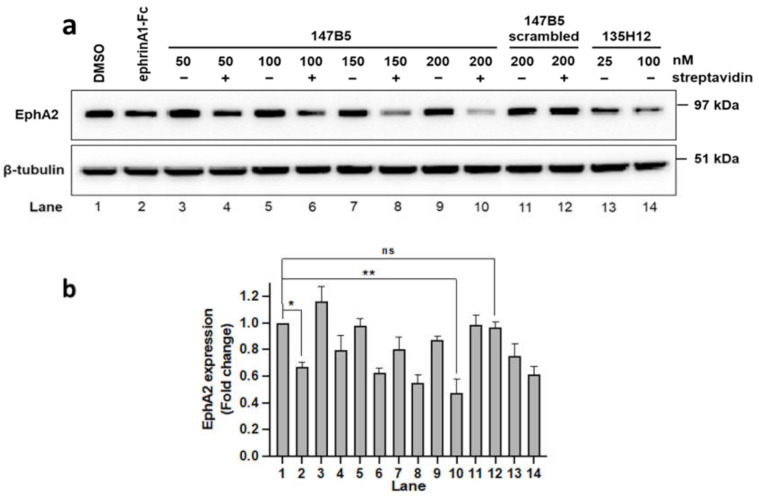
Effect of 135H11-biot (147B5, Table 1), and its complexes with streptavidin at various ratios, on EphA2 receptor degradation. (**a**) Western blot of MDA-MB-231 cells treated (1 h) with ephrinA1-Fc, EphA2 agents 135H12 (135H11 synthetic dimer; tested at 50 nM and 200 nM monomer concentration), and 147B5 (50 nM to 200 nM) and its scrambled-biot corresponding compound (200 nM) (Table 1), tested in the absence and presence of streptavidin (50 nM) for 1 h. Anti-EphA2 blot suggests that the positive control ephrinA1-Fc (22 nM monomer) treatment led to significant degradation of the EphA2 receptor, similar to (147B5)_n_-streptavidin when *n*= 3 or 4 (150 nM or 200 nM 147B5 concentration in the presence of 50 nM streptavidin) but not when *n* = 1. (**b**) Densitometry analysis based on duplicate measurements of band intensities relative to the bands of DMSO-treated cells. * *p* ≤ 0.05, ** *p* ≤ 0.01 as determined by a one-way analysis of variance using Dunnett post-test analysis. Uncropped images for the WB are available as supplementary materials.

We did not expect that the dimer would display increased affinity compared with its monomer for the isolated EphA2-LBD [31,35] (Table 1). Hence, to evaluate the ability of the newly-derived biotinylated version of 135H11 (147B5, Table 1) to activate the receptor, we monitored the ability of the agents to induce EphA2 internalization and degradation in the TNBC cell line MDA-MB-231, and the pancreatic cancer cell line BxPC3. In these cell-based assays, receptor activation and degradation could be simply monitored by detecting the ability of each agent to reduce EphA2 expression levels over time, after exposure of cells to test ligands. We tested various (147B5)*_n_*-streptavidin ratios (*n* = 1, 2, 3, or 4), while dimeric ephrinA1-Fc and 135H12 were used as positive controls. Negative controls were DMSO-treated cells or cells treated with the (scrambled 147B5)_4_-streptavidine complex (Figure 3). 

For the control scrambled agent, we tested it at its maximal 4:1 molar ratio with streptavidin. For these experiments we treated cells with increasing concentrations of 147B5 (from 50 nM to 200 nM) in the absence or presence of streptavidin (50 nM). Positive controls included cells treated with 135H12 (at 50 nM or 200 nM, monomer concentration) or ephrinA1-Fc at 1 μg/mL concentration (~22 nM ephrinA1). 

After a brief exposure time (1 h), cell lysates were probed for total EphA2 using an anti-EphA2 antibody (1C11A12; Thermo Fisher Scientific). When tested against MDA-MB-231 breast-cancer cells (Figure 3), control agents ephrinA1-Fc and 135H12 were very effective in causing EphA2 degradation, compared to a vehicle control. In contrast, monomeric 147B5-streptavidin (1:1 ratio 50 nM) was less effective in causing receptor degradation, in agreement with several previous studies indicating that monomeric agents, including 135H11 alone, showed receptor activation only at very high concentrations (100 μM or higher) [5,29,31,35,36,37]. However, increasing the 147B5/streptavidin ratio resulted in dimeric, trimeric, or tetrameric compounds that induced a more sizable reduction of EphA2 levels in exposed cancer cells (Figure 3). In contrast, the negative control tetrameric (147B5-scrambled)_4_-streptavidin did not alter EphA2 levels (Figure 3). Because receptor activation caused its rapid degradation, we opted to measure total EphA2 levels and not receptor phosphorylation or de-phosphorylation events that would be confounded by overall protein degradation.

**Figure 4:**
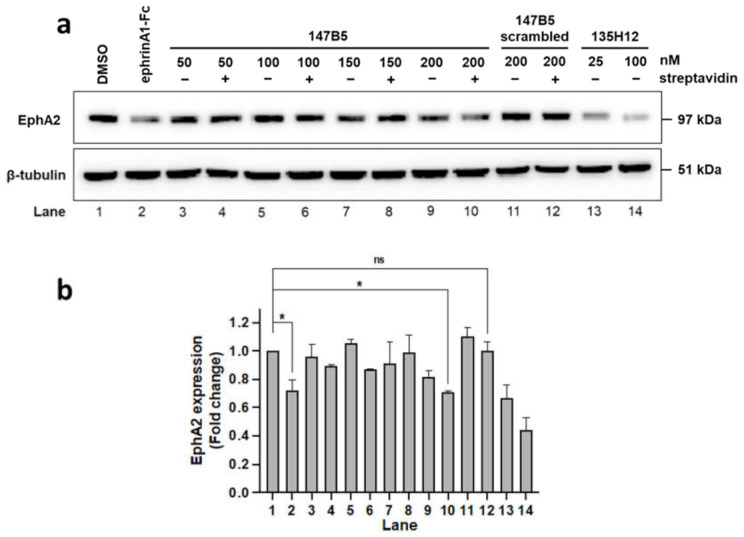
Effect of 147B5 and its complexes with streptavidin at various ratios on EphA2 receptor degradation. (**a**) Western blot study of BxPC3 pancreatic cancer cells treated (1 h) with ephrinA1-Fc, EphA2 agents 135H12 (135H11 synthetic dimer; tested at 50 nM and 200 nM monomer concentration), and 147B5 (50 nM to 200 nM) and its scrambled version (200 nM) (Table 1), tested in the absence and presence of streptavidin (50 nM), as indicated and for 1 h. Anti-EphA2 blot indicates that the positive-control ephrinA1-Fc (22 nM monomer) treatment led to nearly complete degradation of the EphA2 receptor, similar to (147B5)_n_-streptavidin when *n* = 2, 3 or 4 (hence at 100 nM, 150 nM, or 200 nM 147B5 concentration in the presence of 50 nM streptavidin), but not when *n* = 1. (**b**) Densitometry analysis based on duplicate measurements of band intensities relative to the bands of DMSO-treated cells. * *p* ≤ 0.05, as determined by a one-way analysis of variance using Dunnett post-test analysis. Uncropped images for the WB are available as supplementary materials.

In a similar experiment, BxPC3 pancreatic-cancer cells were exposed to the same treatments as described above for MDA-MB-231 cells and total EphA2 level in each treatment was probed (Figure 4). Although multimeric EphA2-targeting agents appeared very effective in both cell lines, the data suggested that degradation of the receptor seemed more pronounced with the BxPC3 pancreatic-cancer cells compared to breast-cancer MDA-MB-231 cells.

These results clearly suggest that the streptavidin–biotin system can be used to increase the agonistic activity of EphA2-targeting ligands such as 135H11. Given the readily-available conjugated forms of streptavidin and the ease of biotinylating other agents, this platform represents a versatile strategy to couple EphA2 targeting agents with other functionalities, including, for example, imaging reagents or chemotherapy as we demonstrate below using orthotopic models of breast and pancreatic cancers. 

### 2.2. (147B5)_4_-Streptavidin-AlexaFluor Visualized in MDA-MB-231 TNBC and BxPC3 Pancreatic Cancer Orthotopic Nude-Mouse Models

**Figure 5:**
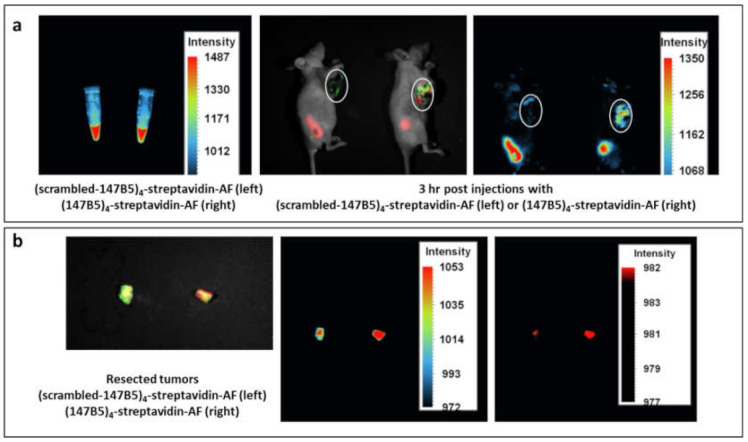
(147B5)_4_-streptavidin-AF accumulates in breast cancer. (**a**) Mice harboring orthotopic human breast cancer MDA-MB-231-GFP (white circles) were treated with either (147B5)_4_-streptavidin-AF or its control (scrambled-147B5)_4_-streptavidin-AF (100 μL, via tail vein, 10 mg/kg) and images were taken at various time points. (**b**) images for the resected tumors from the experiment in (**a**).

To assess the utility of the streptavidin–biotin complex to target EphA2, streptavidin labeled with AlexaFluor was coupled with four equivalents of 147B5 or with its scrambled equivalent. Subsequently, we used orthotopic nude-mouse models of MDA-MB-231 TNBC, expressing green fluorescent protein (GFP). Tumor stock was grown subcutaneously (s.c.) by injecting 5 × 10^6^ MDA-MB-231-GFP cells in 100 μL PBS into the flank of nude mice. The strong GFP expression in the tumors grown in the subcutis of mice was demonstrated using the FluorVivo imaging system (INDEC Biosystems, Los Altos, CA) before tumor harvest. The tumor stock was harvested, inspected and any suspected or grossly necrotic tissues or non-GFP-expressing tumor tissues were removed. Selected tumor tissues were subsequently cut into small fragments of approximately 1 mm^3^ and used for orthotopic tumor implantation in nude mice. Fluorescence whole-body imaging was used to monitor tumor growth. After 21 days, mice were injected with either (147B5)_4_-streptadivin-AlexaFluor or the (scrambled-147B5)_4_-streptavidin-AlexaFluor control (100 μL via the tail vein, to obtain 10 mg/kg) and green and red fluorescence in vivo imaging was used to monitor the EphA2-targeting strategy (Figure 5). Three hours after injection, red fluorescence could be observed that clearly co-localized with green-fluorescence (tumor site) more predominantly in (147B5)_4_-streptadivin-AlexaFluor treatment versus the scrambled agent (Figure 5). This could be appreciated also in the resected tumors, suggesting that EphA2 targeting is accumulating the fluorescent dye at the tumor. 

**Figure 6:**
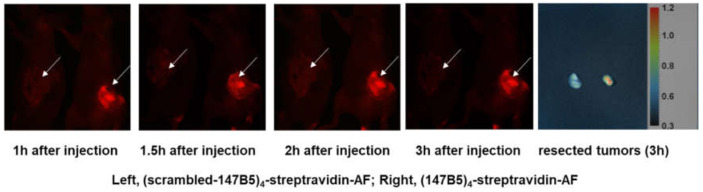
(147B5)_4_-streptavidin-AF accumulates in pancreatic cancer. Mice harboring orthotopic human pancreatic cancer BxPC3-GFP (white arrows) were treated with either (147B5)_4_-streptavidin-AF or its control (scrambled-147B5)_4_-streptavidin-AF (100 μL, via tail vein, 10 mg/kg) and images were taken at various time points.

To extend these findings to pancreatic cancer as well, we used the human pancreatic cancer cell line BxPC-3-GFP. First, tumor stock was grown subcutaneously (s.c.) by injecting 5 × 10^6^ BxPC-3-GFP cells in 100 μL PBS into the flank of nude mice. The strong GFP expression in the tumors grown in the subcutis of mice was demonstrated using the FluorVivo Imaging system before tumor harvest. Viable tumor tissues were subsequently used for orthotopic tumor implantation. Primary tumor size was measured using a fluorescence imaging system up to day 35, at which time mice were injected with either (147B5)_4_-streptadivin-AlexaFluor or the (scrambled-147B5)_4_-streptavidin-AlexaFluor control (100 μL via the tail vein, 10 mg/kg), and green and red fluorescence in vivo imaging was used to assess the EphA2-targeting strategy (Figure 6). Red signal accumulated at the tumor site immediately after injection in the (147B5)_4_-streptadivin-AlexFluor treated versus the scrambled equivalent complex (Figure 6). Concentration of fluorescence in the tumor is also appreciable in the resected tumors (Figure 6), again in agreement with cellular data on EphA2 receptor internalization by (147B5)_4_-streptadivin. 

Collectively, these data strongly suggest that the proposed peptide-biotin-streptavidin system can effectively target EphA2-rich cells in vitro and in vivo, and thus it could find a variety of applications in basic or translational studies to further evaluate whether targeting EphA2 is a viable therapeutic strategy. 

## 3. Materials and Methods

### 3.1. Synthetic Chemistry

For the synthesis of the agents we followed standard solid-phase strategies using a Rink amide resin and a Liberty Blue Peptide Synthesizer (CEM Corp.). All reagents were commercially available including Fmoc (fluorenylmethyloxycarbonyl) protected amino acids, the N-terminal acid, and resins that were used without further purification. The synthetic protocol for each coupling involved 6 equivalents of Fmoc-amino acid, 3 equivalents of N, N’-Diisopropylcarbodiimide (DIC), and 1 equivalent of OximaPure in 4.5 mL of DMF (dimethylformamide) (90 °C, 5 min via microwave irradiation). The following step involved Fmoc deprotection that was performed by treatment with 20% piperidine in DMF (2 × 3 mL) (90 °C for 3 min). Final peptide cleavage from the resin was accomplished with a mixture containing TFA (trifluoroacetic acid), triisopropylsilane, water, phenol (94:2:2:2) for 3 h, followed by precipitation of the peptide in cold diethyl ether. After redissolving the precipitate in DMSO, the solution was purified by preparative RP-HPLC using a XTerra C18 column (Waters) with a JASCO preparative HPLC system and gradient water/acetonitrile (5% to 70%) containing 0.1% TFA (purity > 95%). The identity of the peptides was further confirmed by high resolution mass spectrometry. 

### 3.2. Cell Lines, Cell Culture, and Antibodies

BxPC-3 and MDA-MB-231 cell lines were purchased from the American Type Culture Collection (ATCC) and cultured in RPMI-1640 and DMEM, respectively. The media were supplemented with 10% FBS and 1% Pen/Strep. Anti-EphA2 antibody (1C11A12) and HRP-conjugated goat anti-mouse secondary antibody were purchased from Thermo Fisher Scientific and β-tubulin antibody was purchased from Santa Cruz Biotechnology.

### 3.3. Immunoblotting Assays

Cells were lysed with cell lysis buffer (20 mM Tris, pH 7.4, 120 mM NaCl, 1% Triton X-100, 0.5% sodium deoxycholate, 0.1% SDS, 1% IGEPAL, 5 mM EDTA), supplemented with EDTA-free Protease Inhibitor Cocktail and PhosStop (Sigma Aldrich) for 15 min on ice. Cell lysates were then centrifuged to remove cell debris for 20 min at 13,000 rpm at 4 °C. Samples were prepared and loaded into 4–12% NuPAGE Bis-Tris Precast Gels and transferred to PVDF membranes as indicated previously [35]. The membranes were blocked with 5% non-fat milk in TBS and 0.1% Tween (TBST) for 1 h, probed for primary antibodies raised against EphA2 or β-tubulin at 4 °C overnight, and then with secondary antibodies for 1 h. Subsequently, Clarity Western ECL solution (BIO-RAD) was added to the blots prior to being imaged on a FluorChem (ProteinSimple) and analyzed with AlphaView software.

### 3.4. Establishment of MDA-MB-231 and BxPC3 Orthotopic Models of Human Breast Cancer and Pancreatic Cancers, Respectively

Human breast cancer MDA-MB-231-GFP and human pancreatic cancer cell line BxPC-3-GFP were maintained in DMEM supplemented with 10% heat-inactivated fetal bovine serum and 1% penicillin and cultured at 37 °C in a 5% CO_2_ incubator. Tumor stocks were grown subcutaneously (s.c.) by injecting either 5 × 10^6^ MDA-MB-231-GFP cells or 5 × 10^6^ BxPC-3-GFP cells in 100 μL PBS into the flank of nude mice. The strong GFP expression in the tumors grown in the subcutis of mice was demonstrated using the FluorVivo Imaging system before tumor harvest. The tumor stock was harvested, inspected, and any suspected or grossly necrotic tissues or non-GFP-expressing tumor tissues were removed; tumor tissues were subsequently cut into small fragments of approximately 1 mm^3^ and used for orthotopic tumor implantation. All procedures of the surgery were performed under an 8× magnification microscope under a HEPA-filtered laminar flow hood. Animals were anesthetized by intramuscular injection of ketamine. The surgical area was sterilized using iodine and alcohol. For the breast cancer model, an incision of approximately 0.5 cm long was made in the nude mouse using surgical scissors to expose the second mammary gland. The capsule of the mammary gland at the transplantation site was stripped, and one MDA-MB-231-GFP tumor fragment (1 mm^3^) was transplanted and secured with 8-0 surgical sutures (nylon) [39]. The skin was closed with 5-0 surgical sutures. For the pancreatic cancer model, an incision of approximately 1 cm long was made in the left upper abdomen of the nude mouse using surgical scissors. The pancreas was exposed, the capsule of the pancreas at the transplantation site was stripped, and one BxPC-3-GFP tumor fragment (1 mm^3^) was transplanted and secured with 8-0 surgical sutures (nylon). The abdomen was closed with 5-0 surgical sutures [40]. 

After 21 days (breast cancer) or 35 days (pancreatic cancer), mice received 100 μL, via tail vein, of either (147B5)_4_-streptavidin-AF or (scrambled)_4_-streptavidin-AF and images were acquired using the FluorVivo fluorescence imaging system [41]. Images were processed for contrast and brightness and analyzed with the use of IMAGE PRO IMAGE 6.1 software. High-resolution images of 1392 × 1040 pixels were captured directly on a Lenovo PC. Likewise, after euthanasia, tumors were resected and imaged. 

## 4. Conclusions

Therapeutic targeting of the EphA2-LBD for the development of novel therapeutics in oncology is currently being pursued by a variety of approaches [19,25,32,37,42,43,44,45,46,47,48,49,50]. We recently reported that dimeric 12mer agonistic peptides can be very effective in inducing EphA2 receptor dimerization and subsequent activation and degradation [29,30,31,35]. These previous studies suggested a possible molecular mechanism of EphA2 activation by dimeric agents that involved transient EphA2 dimerization and subsequent clustering and internalization [29,51]. This is well in agreement with previous observations with EphA2 activation by isolated ephrinA1 ligands, which require dimerization or clustering for optimal activity in the cell. In an attempt to create a novel and more flexible EphA2-targeting system, we report here that proper peptide–biotin streptavidin complexes can be used to effectively target EphA2 in both breast- and pancreatic-cancer cells and tumors. The process of receptor dimerization, clustering, internalization, and degradation is complex and not fully understood at the molecular level, given its complexity. However, current theories evoke the possibility that preformed clusters are presumably more prone to activation and degradation, involving secondary interactions between the second extracellular fibronectin domain binding transiently with the ligand-binding domain of an adjacent receptor [38]. Engagement of ephrinA1 ligands in *cis* (within the same cell) could also make the ligand-binding domain less available to external agonistic agents. Speculatively, we propose that these phenomena could attenuate or amplify the effect of agonistic agents in different cancer cell lines, as we observed in the present report comparing the effect of agonistic agents in targeting EphA2 in MDA-MB-231 or BxPC3. 

Given that several streptavidin conjugates are readily available, and considering the ease of synthesis of biotinylated agents, we believe that the proposed system can be useful in devising novel pharmacological tools to evaluate the merits and pitfalls of EphA2 targeting in vitro and in vivo. Hence, we envision that derivatizing streptavidin with imaging reagents and/or cytotoxic agents can result in novel therapeutic agents for diagnostic purposes and/or for targeted delivery of chemotherapy. 

## Figures and Tables

**Table 1:** Sequences of agonistic agents cited in the present report. IC_50_ values (nM) were derived from the DELFIA assay while dissociation constant (Kd) values were obtained by reverse ITC measurements. Reported standard errors represent the indicated number of experiments, each having duplicate measurements. Hyp = trans 4-hydroxy-L-proline. N.B. No binding detected by ITC (see also Figure 1). LC indicates a long 6 atom hydrocarbon chain between the biotin and the amide bond at the C-terminus of the agent.

ID	Sequence	IC_50_ (DELFIA) or K_d_ (ITC) (nM)
135H11	(3-CH_3_,6,7-OCH_3_,Benzofuranoic acid)LA(4-CH_3_-Tyr)PDA V(Hyp)(4Cl-Phe)RP -CONH_2_	130 ± 1, *n* = 4 (DELFIA)
147B5 (135H11-biotinylated)	(3-CH_3_,6,7-OCH_3_,Benzofuranoic acid)LA(4-CH_3_-Tyr)PDA V(Hyp)(4-Cl-Phe)RP-GK(Biotin LC) -CONH_2_	117 (ITC)
135H12 (dimer of 135H11)	((3-CH_3_,6,7-OCH_3_,Benzofuranoic acid)LA(4-CH_3_-Tyr)PDAV(Hyp)(4Cl-Phe) RPG)_2_-K-CONH_2_	150 ± 60, *n* = 3 (DELFIA)
Scrambled-135H11	(3-CH_3_,6,7-OCH_3_,Benzofuranoic acid)DP(4-CH_3_-Tyr)A(Hyp)LRG(4-Cl-Phe)PVA-CONH_2_	>10,000 (DELFIA)
Scrambled-147B5 (scrambled 135H11-biotinylated)	(3-CH_3_,6,7-OCH_3_,Benzofuranoic acid)DP(4-CH_3_-Tyr)A(Hyp)LRG(4-Cl-Phe)PVA-GK(Biotin LC)-CONH_2_	N.B. (ITC)

## Data Availability

Raw data relative to the western blots are available as supporting information.

## Supplementary Materials

The following are available online, Uncropped western blot images (MDA-MB-231 and Bx-PC3).

## Sample Availability

Samples of the compound 147B5 are available from the authors for research purposes and upon signing a standard MTA.
