# Supplementary figures and images for: Effective Tumor Targeting by EphA2-Agonist-Biotin-Streptavidin Conjugates
Source: Molecules. 2021 Jun 17;26(12):3687. doi: 10.3390/molecules26123687 (PMC8235110; doi:10.3390/molecules26123687)

UNCROPPED WESTERN BLOTS

MDA-MB-231

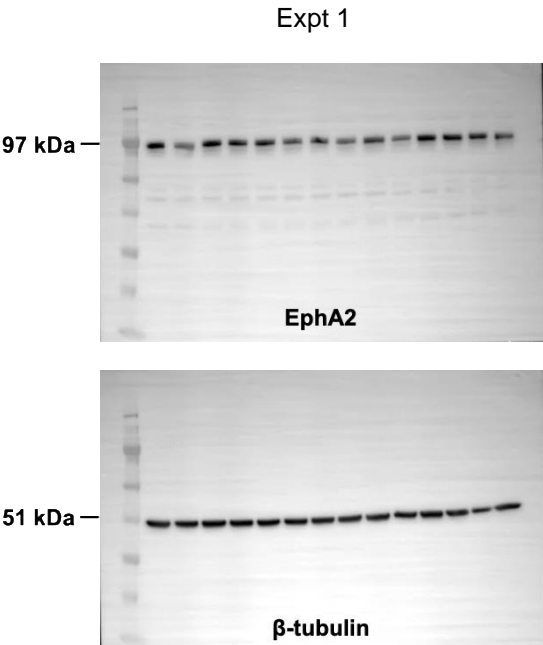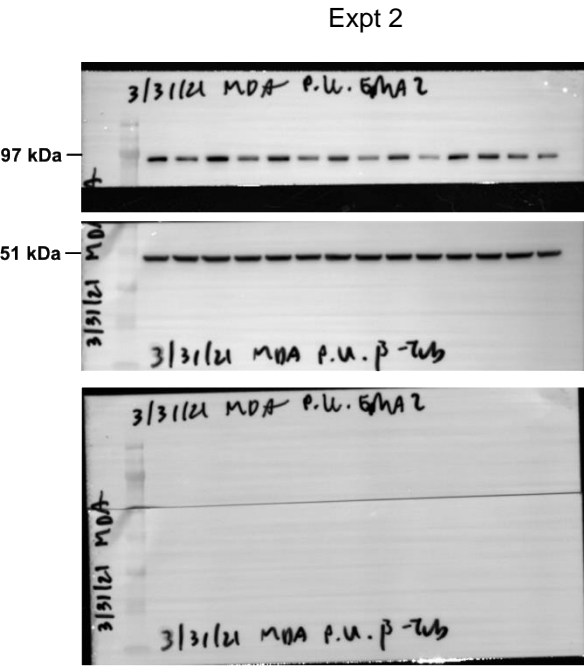

Bx-PC3

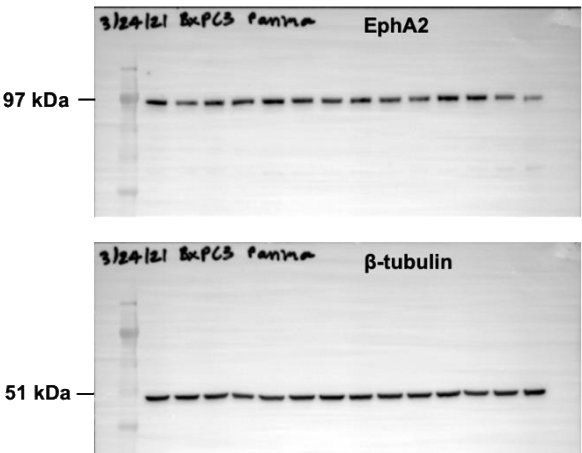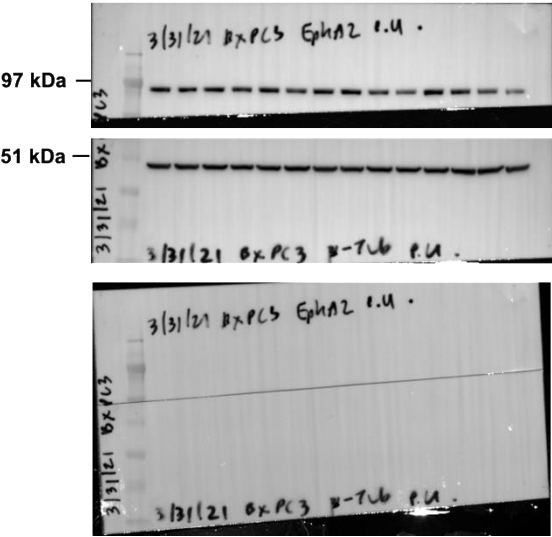

Supplement: Supplementary file 1 [file molecules-26-03687-s001.zip › molecules-1243216-supplementary.pdf]
